# Household Registration System, Migration, and Inequity in Healthcare Access

**DOI:** 10.3390/healthcare7020061

**Published:** 2019-04-11

**Authors:** Bocong Yuan, Jiannan Li, Zhaoguo Wang, Lily Wu

**Affiliations:** Faculty of Economics and Management, Sun Yat-sen University, West Xingang Rd. 135, Guangzhou 510275, China; yuanbc@mail.sysu.edu.cn (B.Y.); wull9@mail2.sysu.edu.cn (L.W.)

**Keywords:** inequity, healthcare access, rural–urban disparity, household registration system, healthcare resource allocation, migration

## Abstract

This study investigates the influence of the household registration system on rural–urban disparity in healthcare access (including healthcare quality, blood pressure check, blood test, vision test, dental examination, and breast exam), using data from a large-scale nationwide life history survey that covered 150 counties across 28 provinces and municipalities in China. In contrast to the findings of many previous studies that emphasize the disparity in the residence place as the cause of rural–urban disparity in healthcare access, this study finds that the residence place just has a very limited influence on healthcare access in China, and what really matters is the household registration type. Our empirical results show that people with a non-rural household registration type generally have better healthcare access than those with a rural one. For rural residents, changing the registration type of their household (from rural to non-rural) can improve their healthcare access, whereas changing the residence place or migrating from rural to urban areas have no effect. Therefore, mere rural-to-urban migration may not be a valid measure to eliminate the rural–urban disparity in healthcare access, unless the institution of healthcare resource allocation is reformed.

## 1. Introduction

Rural–urban disparity in healthcare access is especially reflected in the ease of physician visit, rehabilitative and palliative healthcare services, emergency health service, etc. [1,2,3]. It can lead to further rural–urban disparity for various health-related risk factors such as mortality, morbidity, overall well-being, and lifestyle behaviors [4]. Most studies argue that the social economic discrepancy between residence places is the most important reason for rural–urban disparity in healthcare access. Insufficient healthcare facilities, shortage of experienced healthcare practitioners, disadvantaged economic status, and lack of basic health insurance coverage restrict the access to adequate healthcare for rural residents [5,6,7,8,9,10]. In view of this, a number of studies propose that advancing urbanization or migration from rural to urban areas can improve healthcare access for rural residents to some extent [11,12]. However, the utilization of healthcare resources in urban areas is still very limited for rural-to-urban migrants in China, and changing the residence place from rural to urban areas seems to have a very limited effect on the improvement of healthcare access [13,14,15]. As such, the deep-rooted reason for rural–urban disparity in healthcare access still needs deep investigation.

In China, a number of rural-to-urban migrants adopt self-medication or refuse to seek healthcare services even though they are in bad health [14]. Although the more advanced healthcare facilities in urban areas are open to everyone including rural-to-urban migrants, the utilization of those facilities by the latter is still limited, because of the lack of basic health insurance coverage [14,15,16]. In China, rural-to-urban migrants cannot unconditionally enjoy basic health insurance that benefits urban residents [17,18,19]. Rural-to-urban migrants can only continue benefiting from their original rural basic health insurance, whose extent of coverage is more limited than that of the urban one [20]. Moreover, as required by the principle of dependency coverage, rural-to-urban migrants can benefit from their basic health insurance only within their area of origin, thus they cannot be covered unless they return to their hometown to seek healthcare services [21,22,23]. 

From this point of view, the institution of healthcare resource allocation can be the culprit of the rural–urban disparity in healthcare access. The separation or asynchronization between the residence place and health insurance coverage further solidifies this disparity and makes it persist among rural-to-urban migrants. The institution that plays an important role in determining the status of basic health insurance is the household registration system (also called hukou in Chinese). Since 1955, to restrict internal population migration, the government of China has set up a household registration system, the so-called rural–urban dual hukou system [24,25,26]. The system records the residential location of every citizen and divides registered residents into rural and urban (non-rural) groups. People who live in cities hold non-rural household registration, whereas farmers and other rural residents hold rural household registration. The restrictions related to the household registration system can create disparities in healthcare access in China. Specifically, people with non-rural and rural household registration status in China are covered by separate health insurance systems. People with non-rural household registration will have the urban health insurance (including the employee-based health insurance designed for urban employed population or retirees, and the urban resident health insurance designed for non-employed non-rural household registration holders), while those with rural household registration are allowed to have the rural health insurance which is called New Cooperative Medical Scheme (NCMS) and is operated at the county level. Compared with the rural health insurance, however, the urban health insurance can offer better health coverage, protection, and reimbursement [20,27,28]. Further, most of the health resources in China are usually out of reach for NCMS enrollees and are concentrated in urban China (for example, there were only 1.4 physicians per 1000 people in rural China in 2012, which contrasted sharply with the 3.9 physicians per 1000 people in urban China). Thus, the non-rural household registration can offer people more and better access to health care. 

Moreover, with the rapid development of urbanization in the past three decades, China has seen the largest human migration in history and there has been a large influx of migrant workers from the rural areas into the urban areas [29,30,31,32,33]. The rural-to-urban migrants, even if residing in urban areas, still hold a rural household registration. Switching official residency to a non-rural household registration remains difficult for them. Their opportunities are generally limited into formal employment by state-owned enterprises, enrollment in colleges or universities, demobilization from military services, and promotion to senior administrative jobs. Thus, the current household registration system may create a serious barrier to healthcare services for rural-to-urban migrants, because of their rural household registration. They are usually regarded as temporary employees in urban areas and are not entitled to many public goods, welfare benefits, and community services available to urban residents. Even though they are entitled to NCMS benefits, they are unable to access them in urban areas [16,34] and can obtain them only in the rural areas where their original communities are located. Statistical results from a large study conducted in Shenzhen, one of the major destinations for rural-to-urban migrant workers, showed that more than half of migrant workers (55%) were uninsured [35]. As a consequence of the restriction created by the household registration system, rural-to-urban migrant workers have also been found to consistently underuse health services, both in their original areas and in their destination areas [36,37]. In addition, the household registration status of migrant workers also affects healthcare access for their children. Since children inherit the household registration status from their parents, those with rural household registration are restricted to use NCMS benefits within their home county that offers fewer healthcare resources, independently of whether they still live there or migrated to urban areas with their parents. In light of the high frequency of job mobility of migrant workers, the health coverage for their children is less extensive not only than that of urban children but also than that of rural non-migrant children [38,39,40,41]. Therefore, even though some efforts have been devoted by the government (e.g., basic health-care services through community health-service centers for migrant workers implemented in Beijing) [42,43], progress in the improvement of urban healthcare access for migrant workers has been slow across China. The household registration system can create a serious barrier to healthcare access for rural household registration holders, leading to the existing great disparities in healthcare access between urban and rural areas in China [44,45,46]. 

This study contributes to the existing literature in several aspects. First, this study advances the research on rural–urban disparity in healthcare access by exploring the institutional factor. Second, although the relation between household registration system and healthcare access has theoretical importance, empirical analyses of this issue are still rare. To fill this research gap, this study investigates the relation between the institutional factor and rural–urban disparity in healthcare access. Third, this study clarifies the limited effect of the residence place on healthcare access. By investigating different groups of people (rural-to-urban migrants that experience household registration change, rural residents that never migrate, rural-to-urban migrants that never change household registration), this study differentiates the respective impacts of the household registration and residence place on healthcare access. In this way, this study also explains why rural-to-urban migrants still have poor healthcare access, even though they have migrated to urban areas.

## 2. Methods and Materials

### 2.1. Study Design: CHARLS-2014 (The Life History Survey)

The data used in this study came from a nationwide stratified random sampling survey (China Health and Retirement Longitudinal Study—The life history survey, CHARLS-2014). This widely used nationwide survey aimed to collect a high-quality and nationally representative sample of Chinese residents over the age of 45 [47]. The survey was jointly initiated by the Center for Social Science Survey in China and the Youth League Committee of Peking University and was implemented by the National Development Institute of Peking University (Beijing, China). The data of the life history survey [48] were made public in 2016. This life history survey covered 150 counties including 450 communities and villages across 28 provinces and municipalities in China, among which about 52.6% were rural areas and 47.4% were urban areas. The survey covered 12,400 households in total.

### 2.2. Measurement

#### 2.2.1. Dependent Variables

The dependent variables of this study included several aspects of primary healthcare access, that is, healthcare quality, blood pressure check, blood test, vision test, dental examination, breast exam (female only). Details about dependent variables are shown below.

*Healthcare quality*. Respondents were asked “Can you remember the first time you got to see a doctor? What was the type of the doctor?”. 

*Blood pressure check*. Respondents were asked “Did you have your blood pressure checked after you were 16 years old?”.

*Blood test*. Respondents were asked “Did you have your blood tested after you turned 16 years of age, for example, for measurements of cholesterol or blood sugar?”. 

*Vision exam*. Respondents are asked “Did you have a vision test after you turned 16 years of age?”.

*Dental examination*. Respondents were asked “Did you have dental examinations after you turned 16 years of age?”. 

*Breast exam (female only)*. Respondents were asked “Did you have a breast exam after you turned 16 years of age?”. 

#### 2.2.2. Household Registration and Migration Information

*Household registration type.* Respondents were asked “What was your first household registration type?”. 

*Registration type change of household.* Respondents were asked “Has the registration type changed for your first household change?”. 

*Registered household residence change.* Respondents were asked “Has the registered household residence changed for your first household change?”. 

*Residence after migration.* Respondents were asked “After your first migration, were you living in a rural or urban area?”. 

#### 2.2.3. Control Variables

Following previous literature, we also controlled a series of variables that can affect healthcare access. More details are shown below.

*Gender difference*. In light of the noticeable gender inequity in healthcare access, the effect of gender was controlled in this study. One important reason is the social economic disadvantage of females. The other important reason is that traditional values, social culture, or religious beliefs in some countries can hinder females from seeking healthcare services, especially gynecological and reproductive health services which can make them feel embarrassed or humiliated [49,50,51]. 

*Educational level*. Considering that a lower education level was shown to be associated with lower health literacy, which can prevent people from timely utilizing healthcare services [52,53,54], we controlled the effect of the educational level.

*Economic condition*. As a better economic condition can help people access better healthcare services, we controlled the effect of the economic condition. 

*Ethnic minority*. We controlled the effect of belonging to an ethnic minority, since previous studies showed that healthcare services can be comparatively restricted for ethnic minority people because of a variety of barriers such as linguistic incompetence, cultural inadaptation, location or settlement disadvantage, “newness” or user ignorance [55,56,57]. 

*Religious belief*. We controlled the effect of religious beliefs, since previous studies showed that religious belief can serve as an important barrier to healthcare access. Specifically, Muslim women feel more constraints to accessing healthcare because of the religious obligation to maintain bodily sanctity [58,59,60]. Moreover, some culturally and religiously inappropriate healthcare practices can also serve as disincentives for Muslim women to seek for healthcare services [58,59,61]. 

*Whether or not to have health insurance coverage*. We controlled whether or not the respondents had any medical insurance, since previous studies showed that health insurance coverage was closely associated with healthcare access, and uninsured people were more likely to suffer from insufficient healthcare services [60]. 

### 2.3. Grouping Differences and Correlations

We matched the data and constructed a dataset that contained the above-mentioned variables with non-missing observations. The overview of variables (grouping) and correlation matrix of core variables are respectively displayed in Table 1 and Table 2. 

The results of an independent sample *t*-test (Table 1) indicated that the mean values of most variables differed significantly between respondents with rural household registration type and non-rural household type (healthcare quality, *t*-value = 33.9689; blood pressure check, *t*-value = 14.9594; blood test, *t*-value = 16.6747; vision test, *t*-value = 27.2839; dental examination, *t*-value = 20.1783; breast exam (female only), *t*-value = 19.3437; all the above *t*-values are significant at *p*-value < 0.01). 

The results of pairwise correlation matrix (Table 2) showed that the household registration type (rural vs non-rural type) was significantly associated with various aspects of healthcare access (healthcare quality, −0.2394; blood pressure check, −0.1091; blood test, −0.1199; vision test, −0.1913; dental examination, −0.1425; breast exam (female only), −0.1873; all the above correlation coefficients are significant at *p*-value < 0.01). These results of independent sample t-test and pairwise correlation matrix provide preliminary support for the proposition of this study. 

### 2.4. Statistical Analysis

This study used the multiple linear regression method to examine the relations between household registration, migration, and healthcare access. Linear regression is a widely used approach for modeling the relationships between a scalar dependent variable y and one or more independent variables denoted x. The least-square method is generally used to fit the linear regression model. To control the heterogeneity of individuals across different communities and villages, the estimated standard errors were clustered on community/village level. The least-square method was used for the estimation, and the statistical software Stata 13.1 was used in the data analysis. More details are shown below.

The first set of regressions (see Table 3) showed the main results of interest in this study. In this set of regressions, the effects of household registration type (rural type vs non rural type) on healthcare access (healthcare quality, blood pressure check, blood test, vision test, dental examination, breast exam (female only)) were examined. 

In the second set of regressions (see Table 4), this study examined the effects of household registration change and migration on healthcare access for rural residents and revealed which of these factors may play a critical role in improving rural–urban disparity to health access. It is worth clarifying three concepts. Registered household residence change indicates “the change of registered household residence (from one place to another)”. Migration indicates “the change of residence place”, which is the prerequisite for, but does not necessarily lead to, registered household residence change. Change of registration type of household indicates “the change of household registration type (from rural to non-rural type, vice versa)”. The urbanization of the residence place or the migration between different types of areas (from rural to urban areas or urban to rural areas) is the prerequisite for, but does not necessarily lead to, the change of household registration type. In this set of regressions, these three independent variables were entered step by step to clarify their respective influence on healthcare access.

In the third set of regressions (see Table 5), this study examined the effect of household registration type change on healthcare access for rural residents that never migrated to other area, and further extracted the role of household registration type change in improving rural–urban disparity in health access. As mentioned before, these rural residents largely benefit from the urbanization, since they are eligible to change their registered type of household without having to experience migration from rural to urban areas. 

In the fourth set of regressions (see Table 6), this study examined the effect of migration on healthcare access for rural residents that have never changed household registration and further confirmed that migration cannot improve rural–urban disparity in healthcare access, but the institution of household registration has the main role in this. This group of people is the representative of rural-to-urban migrant workers that live and work in cities while keeping their hometown household registration. This regression result can also be illuminating for the understanding healthcare access under the circumstance of separation of household registration and residence.

## 3. Empirical Results

### 3.1. Descriptive Statistics of the Variables

#### 3.1.1. Dependent Variables

*Healthcare quality*. In total, 19,367 answers to the question “Can you remember the first time you got to see a doctor? What was the type of the doctor?” were obtained; 2191 (11.31%) were “general hospital”, 297 (1.53%) were “specialized hospital”, 2814 (14.53%) were “health clinics in towns”, 4678 (24.15%) were “community health station”, 1414 (7.30%) were “private clinic”, 2460 (12.70%) were “without qualification”, 4445 (22.95%) were “barefoot doctor”, 325 (1.68%) were “others”, 743 (3.84%) were “never see a doctor”.

*Blood pressure check*. Of the total 18,944 answers to the question “Did you have blood pressure checked after you turned 16 years of age?”, 13,533 (71.44%) were “yes”, 5411 (28.56%) were “no”.

*Blood test*. In total, 19,445 answers to the question “Did you have your blood tested after you turned 16 years of age, for example, measurements of cholesterol or blood sugar?” were obtained, of which, 10,863 (55.87%) were “yes”, 8562 (44.13%) were “no”.

*Vision exam*. In total, 199,98 answers to the question “Did you have a vision test after you turned 16 years of age?” were collected, of which, 6844 (34.22%) were “yes”, 13154 (65.78%) were “no”.

*Dental examination*. In total, 20,038 answers to the question “Did you have dental examinations after you turned 16 years of age?” were collected, of which, 5130 (25.60%) were “yes”, 14908 (74.40%) were “no”.

*Breast exam (female only)*. In total, 10,512 answers to the question “Did you have a breast exam after you turned 16 years of age?” were collected, of which, 2410 (22.93%) were “yes”, 8102 (77.07%) were “no”.

#### 3.1.2. Household Registration and Migration Information

*Household registration type.* In total, 19,999 answers to the question “What was your first household registration type?” were obtained; 18015 (90.08%) were “rural household registration (i.e., agriculture type)”, 1984 (9.82%) were “non-rural household registration (within which, there were 1818 (9.09%) belonging to the non-agricultural type, and 166 (0.83%) belonging to the unified residence type)”.

*Registration type change of household.* In total, 5640 answers to the question “Has the registration type changed for your first household change?” were collected; 2657 (47.11%) were “yes”, 2983 (52.89%) were “no”.

*Registered household residence change.* In total, 5640 answers to the question “Has the registered household residence changed for your first household registration change?” were collected; 3950 (70.04%) were “yes”, 1690 (29.96%) were “no”.

*Residence after migration.* In total, 7108 answers to the question “After your first migration, were you living in rural or urban areas?” were obtained; 5930 (83.43%) were “living in a rural area”, 136 (1.91%) were “mainly living in a rural area”, 19 (0.27%) were “Time living in rural and urban areas was roughly the same”, 64 (0.90%) were “mainly living in an urban area”, 959 (13.49%) were “living in an urban area”.

#### 3.1.3. Control Variables

*Gender difference*. In total, 20,402 answers were collected, of which, 9682 (47.46%) were “male”, 10,720 (52.54%) were “female”.

*Educational level*. In total, 20,393 answers were obtained; 5 (0.02%) were “Ph. D”, 15 (0.07%) were “master”, 185 (0.91%) were “four-year college”, 387 (1.90%) were “three-year college”, 596 (2.92%) were “vocational school”, 1635 (8.02%) were “high school”, 5257 (25.78%) were “middle school”, 12313 (60.38%) were “primary school and below”.

*Economic condition*. In total, 20,248 answers were obtained; 250 (1.23%) were “a lot better off than them”, 1742 (8.60%) were “somewhat better off than them”, 10,305 (50.89%) were “same as them”, 3219 (15.90%) were “somewhat worse off than them”, 4732 (23.37%) were “a lot worse off than them”.

*Ethnic minority*. In total, 20,412 answers were obtained; 18399 (90.14%) were “not ethnic minority”, 2013 (9.86%) were “ethnic minority”. 

*Religious belief*. In total, 20,418 answers were collected; 17727 (86.82%) were “no religious belief”, 2691 (13.18%) were “have religious belief”. 

*Whether or not have health insurance coverage*. In total, 19,379 answers, of which, 18690 (96.44%) were “have medical insurance”, 689 (3.56%) were “no medical insurance”. 

### 3.2. Results of the Relation between Household Registration Type and Healthcare Access

Table 3 reports the main regression results of this study. The impacts of household registration (hukou) type on many kinds of healthcare access were examined. The results show that the household registration type was significantly associated with healthcare access. More specifically, residents with rural household registration type generally had worse healthcare access compared with those with non-rural household registration type (−1.6429, healthcare quality; −0.1181, blood pressure check; −0.1481, blood test; −0.2269, vision test; −0.1701; dental examination; −0.1799, breast exam; all estimates with *p*-value < 0.01).

### 3.3. Results of the Relations between Household Registration Change, Migration, and Healthcare Access for Rural Residents

The regressions of Table 4 examine the effects of household registration change and migration on healthcare access for rural residents. It is worth mentioning again that the household registration change included the change of the registration type (change from rural to non-rural) and the change of the registered household residence (between different locations and places). The results show that the change of household registration type (from rural to non-rural) was significantly associated with better healthcare access (0.2647, *p*-value < 0.05, healthcare quality; 0.1040, blood pressure check; 0.1451, blood test; 0.2845, vision test; 0.1763, dental examination; 0.1840, breast exam; all with *p*-value < 0.01). Besides, the relationship between the registered household residence change (from one place to another place) and healthcare access was insignificant (0.2183, healthcare quality; −0.0176, blood pressure check; 0.0002, blood test; 0.0199, vision test; 0.0415; dental examination; −0.0011, breast exam; all above estimates with *p*-value > 0.10). Also, the association between migration and healthcare access was generally insignificant (0.0124, healthcare quality; −0.0053, blood pressure check; −0.0144, vision test; −0.0145; dental examination; all above estimates with *p*-value > 0.10; −0.0176, *p*-value < 0.10, blood test; −0.0329, *p*-value < 0.50, breast exam).

### 3.4. Results of the Relation between Household Registration Type Change and Healthcare Access for Rural Residents that Never Migrated to Other Areas

The results of Table 5 suggest rural residents that never migrated to other areas can still get an improvement in their healthcare access by changing the registration type of household (from rural to non-rural). As shown in Table 5, the household registration type change of rural residents that never migrated to other areas was significantly associated with healthcare access (0.5400, *p*-value < 0.01, healthcare quality; 0.0877, *p*-value < 0.05, blood pressure check; 0.1070, *p*-value < 0.01, blood test; 0.0941, *p*-value < 0.01, vision test; 0.0950, *p*-value < 0.01, dental examination; 0.0419, *p*-value < 0.10, breast exam).

### 3.5. Results of the Relation between Migration and Healthcare Access for Rural Residents that Never Changed Household Registration 

Lastly, the results of Table 6 suggest that the rural residents who did not change household registration could not get an improvement in their healthcare access through migration. More specifically, the association between residence after migration and healthcare access was insignificant (0.0064, healthcare quality; 0.0073, blood pressure check; −0.0040, blood test; 0.0115, vision test; 0.0187, breast exam; all above estimates with *p*-value > 0.10; and 0.0192, *p*-value < 0.10, dental examination).

## 4. Discussion

The empirical findings of this study show that, compared with other countries and regions across the world, the household registration (hukou) system unique to China, rather than the residence place, leads to Chinese rural–urban disparity in healthcare access. This is why the rapid development of urbanization in China has not necessarily solved the rural–urban disparity in healthcare access. This study finds that the change of household registration type from rural to non-rural plays a decisive role in improving healthcare access for rural-to-urban migrants. In this way, they can be eligible to enjoy a broader health insurance coverage. The investigation of different groups (rural-to-urban migrants that changed household registration and rural-to-urban migrants that never changed household registration) helped further confirm that the mere change of residence place from rural areas to urban areas cannot unconditionally improve healthcare access for migrants, unless they can simultaneously change their household registration type from rural type to non-rural type. 

Moreover, the investigation of rural residents that never migrated revealed that, although residing in rural areas, they could still get an improvement in healthcare access by changing household registration from rural type to non-rural type, which further confirms the decisive role of household registration type in healthcare access. Meanwhile, this finding also reflects the dilemma of urban villages in China. The savage development of urbanization in China is making the urban areas expand quickly. As a result, those rural areas originally near a city become gradually surrounded by the expanding city and are thus called “urban villages”. In this case, even though the rural residents who live in urban villages already resided inside the city, they still hold the rural-type household registration and thus are legally identified as rural residents. This dilemma leaves quite a large number of rural residents in “urban villages” being denied coverage by the urban basic health insurance, keeping their healthcare access poor. As such, for the great number of rural residents in “urban villages” in China, changing of household registration type from rural type to non-rural means that they can access broader healthcare services that are easily available. 

Although this is a tricky matter, and a long process is required to reform the current household registration system in China, fortunately, some of the existing literature provide some suggestions to solve the rural–urban disparity in healthcare access. For example, the successful experience of the AiCare platform [62], a regional out-of-hospital integrated care platform in Europe, can be used for reference in China. Compared with public/national health systems that feature a “Top-Down” approach, the AiCare platform acts as an effective “Bottom-Up” solution. It is built in collaboration by the private sector, NGOs, and the public sector. It shifts the classical paradigm of care provision to a single patient-centric model and provides a smart, technology-based affordable solution to respond to the needs for care of a wide range of patients. The usage of this platform can therefore provide significant benefits to the patients, with the care customized to fit the patients at best, at lower costs, and with easy access via a user-friendly online environment. 

It may be a feasible to build a similar collaborative health portal platform in China. It emphasizes the centrality of the patient, without differentiating rural and urban residents. Also, its affordable care services consider the comparatively lower economic status of Chinese rural residents and rural-to-urban migrants. With the large diffusion of smartphones in China, such online platforms are expected to be an important channel for accessing healthcare and, to some extent, can help narrow the rural–urban disparity in healthcare access. 

This study is not free of limitations. On the one hand, the nationwide survey used in this study was focused on the traditional and mainstream channels for accessing healthcare and did not consider other emerging and technology-based channels, like online telemedicine service platforms operated by the private sector. Thus, this study could not take into account such information related to healthcare access. Future research can use new data to investigate relevant issues to complement our analysis. On the other hand, because of the type of data available, only a few kinds of primary healthcare access were explored in this study. As more relevant data become available, future research can make more comprehensive explorations. 

## 5. Conclusions

A large influx of rural-to-urban migrant workers has become a striking phenomenon in China as a result of the rapid urbanization that occurred in the past three decades. However, rural-to-urban migration cannot eliminate Chinese rural–urban disparity in healthcare access. The deficiency in healthcare access for this group of people has become an obvious and burning question in China. This study empirically finds that the root cause of Chinese rural–urban disparity in healthcare access lies in the household registration system unique to China. This system causes a separation between the residence place and health insurance coverage, which thus seriously restricts healthcare access for rural-to-urban migrant workers in cities. Only the change of household registration type (from rural type to non-rural type) can improve their healthcare access, but the channels for changing household registration type from rural to non-rural are very limited for them. Moreover, the phenomenon of “urban villages” is another striking product of rapid urbanization in China. The local residents of “urban villages” face the dilemma that they are still considered as rural residents and continue suffering from the deficiency in healthcare access, although the villages where they reside are now located within cities. Similarly, only the change of household registration type from rural to non-rural can improve their healthcare access. Therefore, with the savage development of urbanization in China, the disadvantages of the current household registration system have become increasingly apparent. There is a need to reform this system to keep pace with the social development and to improve healthcare access for various groups in China. 

## Figures and Tables

**Table 1:** Overview of the variables (grouping).

Variables	Measurement	Rural Household Registration Type	Non-rural Household Registration Type	Combined		Difference
		Mean	Mean	Mean	S.D.	*t*-value
**Healthcare Access**						
Healthcare quality	Can you remember the first time you got to see a doctor? What was the type of the doctor? (A doctor in 1 = general hospital, 2 = specialized hospital, 3 = health clinics in towns, 4 = community/village health station, 5 = private clinic, 6 = local doctor without practicing location, 7 = doctor without license, 8 = others, 9 = never seen a doctor)	4.9256	3.2132	4.7571	2.1302	33.9689 **
Blood pressure check	Did you have blood pressure checked after you turned 16 years of age? (1 = Yes, 2 = Never)	1.3010	1.1347	1.2849	0.4514	14.9594 **
Blood test	Did you have your blood tested after you turned 16 years of age, for example, measurements of cholesterol or blood sugar? (1 = Yes, 2 = Never)	1.4599	1.2585	1.4404	0.4964	16.6747 **
Vision exam	Did you have a vision test after you turned 16 years of age? (1 = Yes, 2 = Never)	1.6864	1.3799	1.6566	0.4748	27.2839 **
Dental examination	Did you have dental examinations after you turned 16 years of age? (1 = Yes, 2 = Never)	1.5545	1.7644	1.7440	0.4365	20.1783 **
Breast exam	Did you have a breast exam after you turned 16 years of age? (1 = Yes, 2 = Never, Female respondents only)	1.7960	1.5267	1.7706	0.4204	19.3437 **
**Household registration status**						
Household registration (hukou) type	What was your first hukou type? (1 = Rural hukou, 2 = Non-rural hukou (including non-agricultural hukou and unified residence hukou))	1.0000	2.0000	1.0992	0.2989	-
Registration type change of household	Has the registration type changed for your first hukou change? (1 = Yes, 2 = No)	1.4887	1.7205	1.5259	0.4994	−12.8827 **
Registered household residence change	Has the registered household residence changed for your first hukou change? (1 = Yes, 2 = No)	1.3371	1.1212	1.3024	0.4593	13.0457 **
Residence after migration	After your first migration, were you living in rural or urban areas? (1 = living in a rural area, 2 = mainly living in a rural area, 3 = Time living in rural and urban areas was roughly the same, 4 = mainly living in an urban area, 5 = living in an urban area)	1.1600	4.0490	1.5949	1.3892	−92.3630 **
**Control variables**						
Gender	[1 = Male, 2 = Female]	1.5275	1.5040	1.5251	0.4994	1.9845 *
Education	[……, 10 = Master program, 11 = Ph.D program]	3.6094	5.2816	3.7852	2.1770	−35.4797 **
Economic condition	When you were a child before the age of 17, compared to the average family in the same community/village at that time, how was your family’s financial situation? (1 = A lot better, 5 = A lot worse)	3.5514	3.1898	3.5157	0.9816	15.5772 **
Ethnic minority	Do you or does your spouse/partner belong to an ethnic minority? (belongs to a certain nationality other than the Han)	0.0945	0.1341	0.9847	0.2980	−5.6212 **
Religion	Do you or does your spouse/partner believe in religion?	0.1429	0.1302	0.1315	0.3379	−1.5783
Having insurance or not	Do you have any medical insurance? (1 = Yes, 2 = Never)	1.0348	1.0427	1.0356	0.1853	−1.7585 ^†^

*Notes:* The independent *t*-test of mean values was implemented; ^†^ *p* < 0.10, * *p* < 0.05, ** *p* < 0.01.

**Table 2:** Pairwise correlation matrix of variables in main regressions.

Variables	1	2	3	4	5	6	7	8	9	10	11	12	13
**Healthcare access**													
1. Healthcare quality	---												
2. Blood pressure check	0.0514 *	---											
3. Blood test	0.0776 *	0.4722 *	---										
4. Vision exam	0.0889 *	0.2384 *	0.2825 *	---									
5. Dental examination	0.0705 *	0.1620 *	0.2006 *	0.3196 *	---								
6. Breast exam (female only)	0.0811 *	0.1910 *	0.2318 *	0.2609 *	0.1862 *	---							
**Household registration**													
7. Household registration (hukou) type	−0.2394 *	−0.1091 *	−0.1199 *	−0.1913 *	−0.1425 *	−0.1873 *	---						
**Demographic variables**													
8. Gender	−0.0179	0.0258 *	0.0241 *	0.1601 *	0.0130 *	−0.0015	−0.0141	---					
9. Education	−0.0876 *	−0.1354 *	−0.1375 *	−0.2504 *	−0.1333 *	−0.2507 *	0.2434 *	−0.2894 *	---				
10. Economic condition	0.0394 *	0.0565 *	0.0588 *	0.0740 *	0.0624	0.0740 *	−0.1099 *	−0.0499 *	−0.1552 *	---			
11. Ethnic minority	−0.0422 *	0.0340 *	0.0241 *	−0.0080	−0.0022	−0.0031	0.0397 *	0.0058	−0.0067	−0.0166	---		
12. Religion	−0.0261 *	−0.0080	−0.0242 *	0.0101	−0.0190 *	0.0000	0.0111	0.0351 *	−0.0729 *	−0.0116	0.1394 *	---	
13. Having insurance or not	0.0080	0.0486 *	0.0525 *	0.0362 *	0.0336 *	0.0389 *	0.0128	0.0229 *	−0.0514 *	0.0060	0.0060	−0.0004	---

*Notes:* As the number of non-missing observations was different across variables, the sequence length varies across each pair of variables when computing the correlation coefficients; * *p* < 0.01.

**Table 3:** Effect of household registration (hukou) type on healthcare access.

Variables	Dependent Variable: Healthcare Access					
	Healthcare Quality	Blood Pressure Check	Blood Test	Vision Test	Dental Examination	Breast Exam [Female]
**Independent variable**						
Household registration type	−1.6429 ** [0.0709]	−0.1181 ** [0.0123]	−0.1481 ** [0.0162]	−0.2269 ** [0.0170]	−0.1701 ** [0.0159]	−0.1799 ** [0.0214]
**Control variables**						
Gender	−0.1397 ** [0.0288]	−0.0058 [0.0071]	−0.0077 [0.0077]	0.1037 ** [0.0077]	−0.0162 * [0.0064]	---
Education	−0.0463 ** [0.0097]	−0.0238 ** [0.0020]	−0.0263 ** [0.0023]	−0.0400 ** [0.0022]	−0.0214 ** [0.0019]	−0.0389 ** [0.0024]
Economic condition	0.0126 [0.0157]	0.0127 ** [0.0038]	0.0157 ** [0.0038]	0.0176 ** [0.0035]	0.0125 ** [0.0034]	0.0117 ** [0.0045]
Ethnic minority	−0.2080 * [0.0882]	0.0602 ** [0.0222]	0.0558 ** [0.0175]	−0.0023 [0.0150]	0.0110 [0.0133]	0.0010 [0.0167]
Religion	−0.1494 * [0.0625]	−0.0272 * [0.0136]	−0.0531 ** [0.0140]	−0.0054 [0.0114]	−0.0333 ** [0.0118]	−0.0186 [0.0135]
Having insurance or not	0.1280 [0.0919]	−0.1083 ** [0.0218]	0.1213 ** [0.0217]	0.0639 [0.0188]	0.0697 ** [0.0161]	−0.0564 ** [0.0189]
Intercept term	6.8209 ** [0.1647]	1.3504 ** [0.0370]	1.5318 ** [0.0381]	1.7708 ** [0.0357]	1.9218 ** [0.0316]	1.9953 ** [0.0349]
Number of observations	18028	17690	18118	18550	18586	9675
F statistics [*p*-value]	91.32 [0.0000]	52.52 [0.0000]	51.18 [0.0000]	173.52 [0.0000]	49.33 [0.0000]	74.67 [0.0000]

*Notes:* Estimated standard errors were clustered on community level and are reported in bracket; * *p* < 0.05, ** *p* < 0.01.

**Table 4:** Effects of household registration (hukou) change and migration on healthcare access for rural residents.

Variables	Dependent Variable: Healthcare Access											
	Healthcare Quality		Blood Pressure Check		Blood Test		Vision Test		Dental Examination		Breast Exam [Female]	
**Independent variable**												
Registration type change of household	0.2192 ^†^ [0.1158]	0.2647 * [0.1196]	0.1075 ** [0.0193]	0.1040 ** [0.0193]	0.1450 ** [0.0231]	0.1451 ** [0.0236]	0.2804 ** [0.0252]	0.2845 ** [0.0252]	0.1677 ** [0.0231]	0.1763 ** [0.0244]	0.1842 ** [0.0291]	0.1840 ** [0.0295]
Registered household residence change		0.2183 [0.1386]		−0.0176 [0.0201]		0.0002 [0.0280]		0.0199 [0.0291]		0.0415 [0.0308]		−0.0011 [0.0357]
Residence after migration	0.0306 [0.0586]	0.0124 [0.0597]	−0.0066 [0.0086]	−0.0053 [0.0089]	−0.0176 ^†^ [0.0098]	−0.0176 ^†^ [0.0099]	−0.0144 [0.0108]	−0.0160 [0.0109]	−0.0145 [0.0127]	−0.0178 [0.0126]	−0.0329 * [0.0143]	−0.0328 * [0.0146]
**Control variables**												
Gender	−0.0498 [0.0938]	−0.0441 [0.0938]	0.0300 [0.0170]	0.0295 ^†^ [0.0170]	0.0595 ** [0.0207]	0.0595 ** [0.0208]	0.1672 ** [0.0238]	0.1677 ** [0.0239]	0.0576 ** [0.0201]	0.0588 ** [0.0201]	---	---
Education	−0.0406 ^†^ [0.0208]	−0.0405 ^†^ [0.0208]	−0.0159 ** [0.0039]	−0.0159 ** [0.0039]	−0.0206 ** [0.0043]	−0.0206 ** [0.0043]	−0.0302 ** [0.0043]	−0.0302 ** [0.0043]	−0.0178 ** [0.0045]	−0.0178 ** [0.0045]	−0.0403 ** [0.0047]	−0.0403 ** [0.0047]
Economic condition	−0.0402 [0.0434]	−0.0429 [0.0435]	0.0103 [0.0075]	0.0105 [0.0075]	0.0215 * [0.0084]	0.0215 * [0.0084]	0.0041 [0.0085]	0.0038 [0.0085]	−0.0008 [0.0096]	−0.0013 [0.0097]	−0.0008 [0.0100]	−0.0008 [0.0100]
Ethnic minority	−0.1961 [0.1679]	−0.2037 [0.1660]	0.0343 [0.0238]	0.0349 [0.0238]	0.0630 * [0.0309]	0.0630 * [0.0308]	0.0453 ^†^ [0.0257]	0.0446 ^†^ [0.0258]	0.0121 [0.0325]	0.0108 [0.0327]	0.0059 [0.0297]	0.0060 [0.0297]
Religion	−0.4745 ** [0.1345]	−0.4704 ** [0.1343]	0.0043 [0.0225]	0.0041 [0.0224]	−0.0678 * [0.0271]	−0.0678 * [0.0271]	−0.0118 [0.0244]	−0.0114 [0.0245]	−0.0177 [0.0293]	−0.0170 [0.0294]	−0.0086 [0.0318]	−0.0086 [0.0319]
Having insurance or not	0.1040 [0.2278]	0.1074 [0.2269]	0.1148 * [0.0462]	0.1150 * [0.0463]	0.0986 * [0.0502]	0.0986 * [0.0502]	0.1095 * [0.0430]	0.1096 * [0.0429]	0.1422 ** [0.0406]	0.1425 ** [0.0406]	0.0959 * [0.0401]	0.0958 * [0.0401]
Intercept term	4.7012 ** [0.3988]	4.3993 ** [0.4439]	0.9001 ** [0.0759]	0.9239 ** [0.0781]	0.9584 ** [0.0855]	0.9582 ** [0.0954]	0.8495 ** [0.0793]	0.8222 ** [0.0886]	1.2443 ** [0.0789]	1.1872 ** [0.0925]	1.5100 ** [0.0825]	1.5114 ** [0.0944]
Number of observations	2833	2833	2803	2803	2852	2852	2914	2914	2915	2915	1831	1831
F statistics [*p*-value]	2.70 [0.0066]	2.71 [0.0044]	14.72 [0.0000]	13.05 [0.0000]	24.88 [0.0000]	22.35 [0.0000]	95.12 [0.0000]	85.64 [0.0000]	20.38 [0.0000]	18.28 [0.0000]	22.15 [0.0000]	19.40 [0.0000]

*Notes:* The regression sample in this table is residents whose first hukou was rural and changed household registration (either household residence or type). The household registration (hukou) type change indicates the change between rural and non-rural. Estimated standard errors were clustered on community level and are reported in brackets; ^†^ *p* < 0.10, * *p* < 0.05, ** *p* < 0.01.

**Table 5:** Effect of household registration (hukou) type change on healthcare access for rural residents that have never migrated to other areas.

Variables	Dependent Variable: Healthcare Access					
	Healthcare Quality	Blood Pressure Check	Blood Test	Vision Test	Dental Examination	Breast Exam [Female]
**Independent variable**						
Registration type change of household	0.5400 ** [0.1762]	0.0877 * [0.0341]	0.1070 ** [0.0377]	0.0941 ** [0.0333]	0.0950 ** [0.0322]	0.0419 [0.0309]
**Control variables**						
Gender	0.1170 [0.1201]	−0.0009 [0.0257]	0.0028 [0.0274]	0.1271 ** [0.0290]	−0.0381 [0.0268]	---
Education	0.0626 ^†^ [0.0323]	−0.0150 * [0.0063]	−0.0223 ** [0.0069]	−0.0465 ** [0.0061]	−0.0084 [0.0065]	−0.0581 ** [0.0068]
Economic condition	0.1171 * [0.0581]	0.0084 [0.0126]	−0.0060 [0.0134]	−0.0019 [0.0118]	0.0032 [0.0134]	−0.0146 [0.0165]
Ethnic minority	−0.3476 [0.2832]	0.0242 [0.0466]	0.0526 [0.0486]	−0.0535 [0.0462]	−0.0072 [0.0405]	0.1129 * [0.0534]
Religion	−0.4065 ^†^ [0.2125]	−0.0784 * [0.0343]	−0.0760 * [0.0349]	0.0289 [0.0365]	−0.0431 [0.0363]	−0.0006 [0.0434]
Having insurance or not	0.5627 [0.3824]	−0.0070 [0.0717]	−0.0980 [0.0676]	0.0318 [0.0784]	0.0288 [0.0741]	−0.0655 [0.0946]
Intercept term	2.6418 ** [0.6245]	1.1625 ** [0.1090]	1.4328 ** [0.1125]	1.4574 ** [0.1171]	1.6950 ** [0.1172]	1.9875 ** [0.1251]
Number of observations	1400	1384	1420	1448	1462	827
F statistics [*p*-value]	2.88 [0.0000]	3.61 [0.0000]	4.98 [0.0000]	25.67 [0.0000]	2.22 [0.0000]	15.83 [0.0000]

*Notes:* The regression sample in this table is residents whose first hukou was rural and never migrated to other areas. The household registration (hukou) type change indicates the change between rural and non-rural. Estimated standard errors were clustered on community level and are reported in brackets; ^†^ *p* < 0.10, * *p* < 0.05, ** *p* < 0.01.

**Table 6:** Effect of migration on healthcare access for rural residents that never changed household registration (hukou).

Variables	Dependent Variable: Healthcare Access					
	Healthcare Quality	Blood Pressure Check	Blood Test	Vision Test	Dental Examination	Breast Exam [Female]
**Independent variable**						
Residence after migration	0.0064 [0.0703]	0.0073 [0.0129]	−0.0040 [0.0137]	0.0115 [0.0139]	0.0192 ^†^ [0.0106]	0.0187 [0.0165]
**Control variables**						
Gender	−0.1740 ^†^ [0.0916]	0.0119 [0.0208]	0.0331 [0.0232]	0.1753 ** [0.0206]	0.0227 [0.0180]	---
Education	−0.0356 [0.0255]	−0.0206 ** [0.0060]	−0.0198 ** [0.0062]	−0.0297 ** [0.0053]	−0.0070 [0.0050]	−0.0314 ** [0.0058]
Economic condition	−0.0573 [0.0383]	0.0025 [0.0092]	−0.0011 [0.0111]	0.0093 [0.0096]	−0.0005 [0.0088]	0.0014 [0.0117]
Ethnic minority	−0.0843 [0.1589]	0.0947 * [0.0421]	0.0475 [0.0359]	−0.1031 ** [0.0355]	−0.0210 [0.0315]	−0.0192 [0.0438]
Religion	0.0076 [0.1329]	−0.0490 ^†^ [0.0294]	−0.0425 [0.0343]	−0.0042 [0.0273]	−0.0169 [0.0118]	−0.0441 [0.0366]
Having insurance or not	0.0425 [0.2048]	0.1410 ** [0.0536]	0.1174 * [0.0494]	0.0528 [0.0441]	0.0927 ** [0.0343]	0.0699 [0.0426]
Intercept term	5.6121 ** [0.3329]	1.1787 ** [0.0795]	1.3578 ** [0.0857]	1.4330 ** [0.0783]	1.6449 ** [0.0658]	1.8329 ** [0.0701]
Number of observations	2531	2479	2535	2599	2600	974
F statistics [*p*-value]	0.89 [0.5174]	4.99 [0.0000]	4.17 [0.0002]	25.62 [0.0000]	2.69 [0.0098]	6.36 [0.0000]

*Notes:* The regression sample in this table is residents whose first hukou was rural and never changed household registration (neither household residence nor type). Estimated standard errors were clustered on community level and are reported in brackets; ^†^ *p* < 0.10, * *p* < 0.05, ** *p* < 0.01.
