# Erratum: Yuan, B., *et al.* Household Registration System, Migration, and Inequity in Healthcare Access. *Healthcare* 2019, *7*, 61

**DOI:** 10.3390/healthcare8020156

**Published:** 2020-06-03

**Authors:** Bocong Yuan, Jiannan Li, Zhaoguo Wang, Lily Wu

**Affiliations:** 1 School of Tourism Management, Sun Yat-sen University, West Xingang Rd. 135, Guangzhou 510275, China; yuanbc@mail.sysu.edu.cn (B.Y.); wull9@mail2.sysu.edu.cn (L.W.); 2 International School of Business & Finance, Sun Yat-sen University, West Xingang Rd. 135, Guangzhou 510275, China

Some details about author affiliations should to be updated in the article [1]. We sincerely apologize to our editor, all editorial staff, the publisher and our readers for any inconvenience.

Bocong Yuan, Zhaoguo Wang and Lily Wu are currently affiliated to the School of Tourism Management, Sun Yat-sen University.

Jiannan Li is currently affiliated to the International School of Business & Finance, Sun Yat-sen University.

The corrected author list is provided below:

Bocong Yuan ^1^, Jiannan Li ^2,^*, Zhaoguo Wang ^1,^* and Lily Wu ^1^

^1^ School of Tourism Management, Sun Yat-sen University, West Xingang Rd. 135, Guangzhou 510275, China; yuanbc@mail.sysu.edu.cn (B.Y.); wull9@mail2.sysu.edu.cn (L.W.)

^2^ International School of Business & Finance, Sun Yat-sen University, West Xingang Rd. 135, Guangzhou 510275, China. 

* Correspondence: lijnanna@mail.sysu.edu.cn (J.L.); wzglinyi2007@163.com (Z.W.)
